# MODIS Retrieval of Aerosol Optical Depth over Turbid Coastal Water

**DOI:** 10.3390/rs9060595

**Published:** 2017-06-12

**Authors:** Yi Wang, Jun Wang, Robert C. Levy, Xiaoguang Xu, Jeffrey S. Reid

**Affiliations:** 1 Department of Chemical and Biochemical Engineering, The University of Iowa, Iowa City, IA 52242, USA; 2 Center of Global and Regional Environmental Research, The University of Iowa, Iowa City, IA 52242, USA; 3 Interdisciplinary Graduate Program in Informatics, The University of Iowa, Iowa City, IA 52242, USA; 4 NASA Goddard Space Flight Center, Greenbelt, MD 20771, USA; 5 Marine Meteorology Division, Naval Research Laboratory, Monterey, CA 93943, USA

**Keywords:** AOD, coastal water, MODIS, retrieval

## Abstract

We present a new approach to retrieve Aerosol Optical Depth (AOD) using the Moderate Resolution Imaging Spectroradiometer (MODIS) over the turbid coastal water. This approach supplements the operational Dark Target (DT) aerosol retrieval algorithm that currently does not conduct AOD retrieval in shallow waters that have visible sediments or sea-floor (i.e., Class 2 waters). Over the global coastal water regions in cloud-free conditions, coastal screening leads to ~20% unavailability of AOD retrievals. Here, we refine the MODIS DT algorithm by considering that water-leaving radiance at 2.1 µm to be negligible regardless of water turbidity, and therefore the 2.1 µm reflectance at the top of the atmosphere is sensitive to both change of fine-mode and coarse-mode AODs. By assuming that the aerosol single scattering properties over coastal turbid water are similar to those over the adjacent open-ocean pixels, the new algorithm can derive AOD over these shallow waters. The test algorithm yields ~18% more MODIS-AERONET collocated pairs for six AERONET stations in the coastal water regions. Furthermore, comparison of the new retrieval with these AERONET observations show that the new AOD retrievals have equivalent or better accuracy than those retrieved by the MODIS operational algorithm’s over coastal land and non-turbid coastal water product. Combining the new retrievals with the existing MODIS operational retrievals yields an overall improvement of AOD over those coastal water regions. Most importantly, this refinement extends the spatial and temporal coverage of MODIS AOD retrievals over the coastal regions where 60% of human population resides. This expanded coverage is crucial for better understanding of impact of aerosol particles on coastal air quality and climate.

## 1. Introduction

Aerosols are a colloidal system of particles suspended in the atmosphere, and have significant impacts on weather, climate, and human health [1–3]. Because of its global observational coverage, satellite remote sensing has a critical role in quantifying these impacts. Such satellite remote sensing is being used to retrieve aerosol properties such as the aerosol optical depth (AOD), along with a well-characterized uncertainty envelope, at high spatial resolution across the globe. Indeed, since the launch of the Moderate Resolution Imaging Spectroradiometer (MODIS) on the NASA Terra satellite in 1999, AOD retrievals derived by Dark Target (DT) algorithm [4] or Deep Blue (DB) algorithm (land only) [5,6] have been used widely in the research community [7].

One persistent challenge to aerosol remote sensing is retrieval of environmental properties over coastal (or littoral) waters. Variable ocean color, along with the presence of visible sediments and sea floor along coastlines, has strong spectral and spatial variability leading to poorly constrained lower boundary conditions for aerosol retrievals. Yet, retrieving AOD in coastal waters is a much-desired part of new systems characterizing air quality and aerosol radiative effects. As ~60% of the human population lives in coastal areas [8], it is crucial to expand the satellite-remote sensing datasets to include these areas. This study aims to address this observational gap by refining the MODIS DT-ocean algorithm to retrieve AOD over turbid coastal waters.

The MODIS DT-ocean algorithm uses top of atmosphere (TOA) reflectance in six wavelength bands, ranging from 0.55 to 2.1 µm, to simultaneously retrieve AOD and Fine Mode Fraction (FMF) based on a lookup table approach. The lookup table is constructed by assuming aerosol optical properties of four fine aerosol modes and five coarse aerosol modes, coupled with the boundary conditions of molecular scattering, and a rough ocean surface (glitter, whitecaps, foam). Additionally, the lookup table assumes zero water-leaving radiance at all bands except 0.55 µm (at which a fixed water-leaving reflectance of 0.005 is used) [4,9]. The retrieval process searches the lookup table to find the best combination of fine mode and coarse mode aerosol type (out of possibly 20 combinations) such that the AOD and FMF retrievals render the best match between observed and simulated (e.g., those from the lookup table) radiances. However, while the assumption of nearly-zero water-leaving radiance may be suitable for open ocean, it is clearly not applicable to turbid coastal waters. There, the water-leaving radiances can be contributed by reflection of shallow-water sea floor (particularly in blue wavelengths) and either suspended or dissolved particulate matter in the water, especially in green to red wavelengths such as 0.55, 0.66, and 0.86 µm [10]. Hence, as part of the DT-ocean algorithm, the turbid water pixels are masked and not considered for retrieval. The method used for such masking compares the TOA reflectance at 0.55 µm with the expected counterpart from the power-law fitting using the TOA reflectance at 0.47, 1.2, 1.6, and 2.1 µm; if a significant difference (larger than 0.01) is found, the corresponding pixel is masked out for AOD retrieval [8].

**Figure 1:**
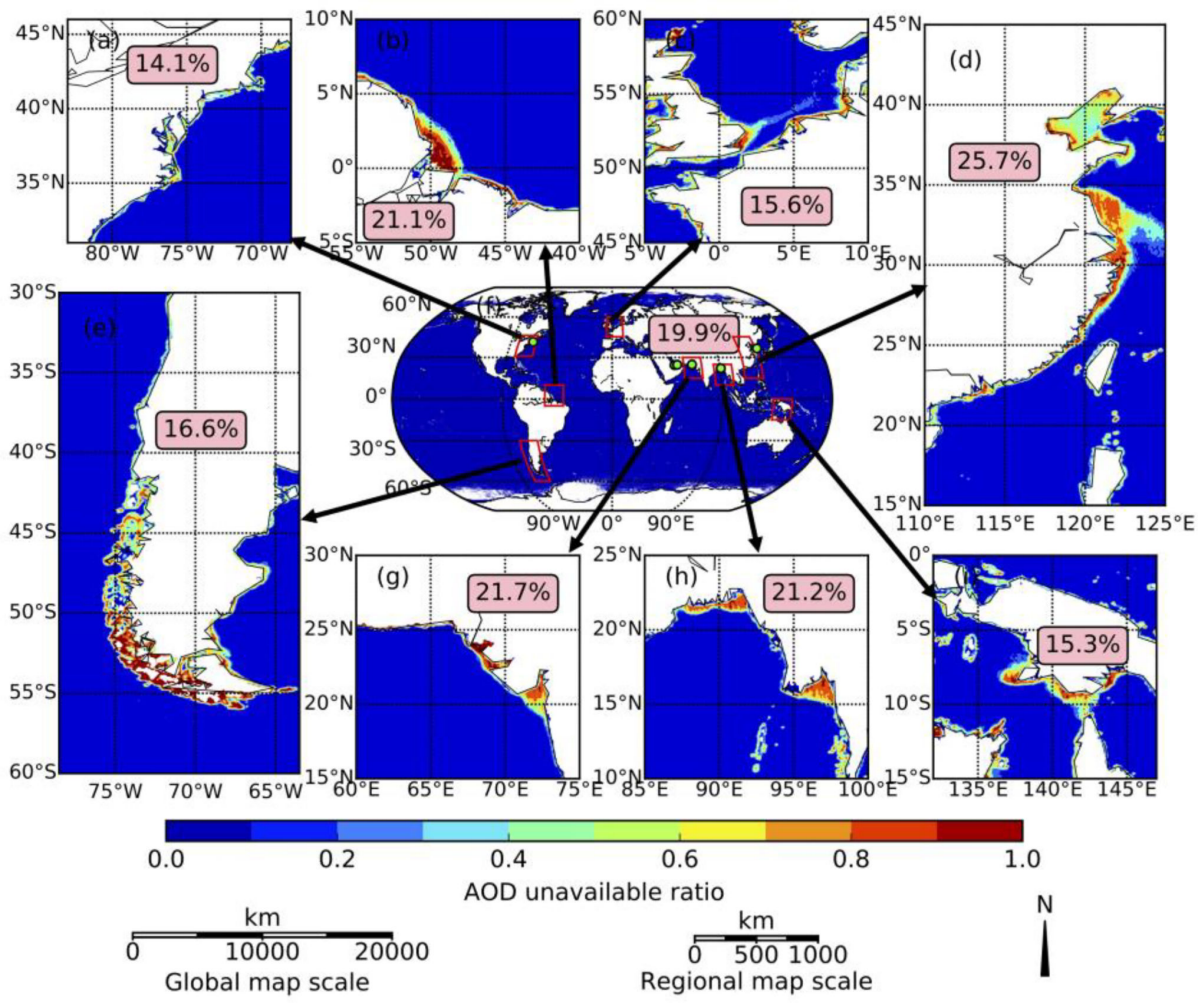
Regional (**a–e** and **g–i**) and global (**f**) distribution of AOD unavailable ratio which is defined as the ratio between number of non-retrieval pixels and all pixels in cloud-free conditions. MYD04 product in 2016 are used to conduct the statistics. Zoom in on the red-box regions of (**f**) are shown in (**a–e** and **g–i**). Lime points in (**f**) are AERONET sites used for validation and more information is in Table 1. Numbers in the pink boxes are AOD unavailable ratio over coastal water in clear sky condition.

We analyze the MODIS AOD retrieval unavailability over the cloud-free conditions at both global and regional scale to reveal how often the AOD is not retrieved only because of water turbidity (and not other factors such as cloud cover). As shown in Figure 1, this data availability is near total over all open ocean and decreases dramatically (by 90–100%) toward coastlines. In a global average, the unavailability of AOD is ~20% over coastal water region which is labelled as shallow ocean (within 5 km of coastline or with water depth less than 50 m) in the MODIS geolocation product [11]. In other words, in these 20% of cloud-free cases, the AOD should have been retrieved if the water was not turbid.

Based on the principle that liquid water absorption increases in shortwave infrared (SWIR), we present a new approach that uses MODIS-measured radiance at 2.1 µm to retrieve AOD over turbid coastal water no matter whether the aerosol is fine or coarse mode dominated. In the spectral range of 0.55 to 2.1 µm, the transparency of pure water to sunlight decreases rapidly with the increase of wavelength; the penetration depth (at which light attenuation is 90%) is 41 m at 0.55 µm and drops to 0.001 m at 2.1 µm [8]. Hence, unless the water is shallower than 1 mm, the water-leaving radiance contributed from the sea floor, sediments, and other contaminants in the water at 2.1 µm is nearly negligible; this vastly simplifies the lower boundary condition of the retrieval.

We present the data and the new approach in Sections 2 and 3, respectively, followed by description of retrieval results and the validation against measurements from six Aerosol Robotic Network (AERONET) coastal sites in Section 4. Discussions and conclusions are in Sections 5 and 6, respectively.

## 2. Data

### 2.1. MODIS Data

MODIS is an earth-viewing sensor on board the Terra and Aqua polar-orbiting satellites, launched in December 1999 and May 2002, respectively. MODIS has 36 channels spanning the spectral range from 0.41 to 15 µm with spatial resolutions of 250 m (2 channels), 500 m (5 channels), and 1 km (29 channels). Its 2330-km swath width enables it to provide near-global converge daily. Terra and Aqua cross the equator from north to south (descending node) at approximately 10:30 a.m. local time and from south to north (ascending node) at approximately 1:30 p.m. local time, respectively.

Here, all MODIS data products are labeled as MxDNN, where x is substituted by O for Terra and Y for Aqua, respectively, and NN is the serial number of a specific product. King et al. [12] presented a general description of MODIS atmosphere data processing architecture and products. In this study, the MODIS-calibrated TOA reflectance product (MxD02) (http://mcst.gsfc.nasa.gov/content/l1b-documents), atmospheric profile product (MxD07), geolocation product (MxD03), and aerosol product (MxD04) [4] are used for retrieving aerosol optical depth over turbid coastal water. TOA reflectance of 2.1 µm with spatial resolution of 500 m from MxD02 is used to retrieve AOD through a lookup table (LUT) method. Gas absorption by water vapor and ozone column are corrected from the TOA reflectance with spatial resolution of 5 km from MxD07 product [4]. The MxD03 product has a spatial resolution of 1 km and is used to distinguish between water and land pixels. All other ancillary information from MxD04 aerosol product for each valid AOD retrieval (at spatial resolution of 10 km at nadir) are also used as input to the retrieval algorithm in this study; the information includes aerosol mode index, reflectance weighting parameter, and National Centers for Environmental Prediction (NCEP) analysis of wind speed (2 m above the surface). In addition, a cloud mask with a spatial resolution of 500 m from MxD04 aerosol product is also used to ensure only AOD during cloud-free conditions over the turbid coastal region is retrieved by this study.

### 2.2. AERONET Data

AOD measurements from the ground-based AERONET sun photometers are commonly used for validating MODIS retrievals. Here we use data from six coastal water sites for this study (Table 1), and their locations are marked with lime points in global map in Figure 1f. All AERONET sun photometers (SP) measure direct solar radiation at 0.44 µm, 0.67 µm, 0.87 µm, 0.94 µm and 1.02 µm, and these measurements are used to infer AOD through Beer-Lambert-Bouguer law with quality at Level 1.0 (unscreened), Level 1.5 (cloud screened), and Level 2.0 (cloud-screened and quality-assured) [13,14]. We evaluate MODIS AOD (both MxD04 product and retrieval of this study) at 0.55 µm against an AERONET counterpart that is derived through linearly interpolating AERONET AOD at 0.44 and 0.67 µm in the logarithm domain.

Dalma and MVCO are two sites over the ocean, with distance to coastline being 48 km and 29 km, respectively, while the rest of the sites are over land within 6 km from the coastline. According to our analysis, most AERONET sites are more than 10 km away from the coast lines, and lack dedicated long-term continuous measurements of AOD over the turbid coastal water. Here, we use the SP AOD data from August 2015, December 2015, May 2016, August 2004, March 2014, and September 2004 at MVCO Bhola, Anmyon, Dalma, Karachi, and MAARCO sites, respectively, because these time periods have the most measurements available at their corresponding sites. AERONET Level 2.0 data are used for most sites except Bhola and Anmyon where only Level 1.5 data are available and utilized here. The monthly mean AERONET 0.44–0.87 µm Ångström exponent ranges from 0.597 to 1.842 over these six sites. Hence, these sites represent a wide range of atmospheric conditions, ranging from coarse mode dominated to fine mode dominated cases [15].

### 2.3. Data Extraction Procedure

MxD04 [4] AOD and the AOD retrieval from the new algorithm are evaluated against AERONET measurements. The spatio-temporal matching approach by Ichoku et al. [16] is applied to collocate AERONET AOD measurements and MODIS retrievals (MxD04 and/or new algorithm) for comparison. AERONET measurements within ±30 min of the MODIS overpass time are averaged and compared against MODIS retrievals averaged within a 50-km diameter circular region centered over the AERONET sites. MxD04 products include DT, DB, and DT/DB merged retrievals, corresponding to 0.55 µm AOD from Dark Target algorithms (DT-land and DT-ocean algorithms), Deep Blue algorithm, and combination of the two retrievals, respectively [4]. DT retrievals cover both vegetated land and ocean while DB is limited to land. As the DT-land algorithm is not designed to retrieve AOD over bright desert, there are significant retrieval gaps over land. The DB algorithm was originally designed to complement the gaps, based on the principal that desert pixels are relatively darker in deep-blue bands [5]. However, now DB has been extended to vegetated land surface as well. Thus, for this study, we use the DB/DT merged product (Quality flag = 1, 2, 3 over ocean, and Quality flag = 3 over land) from MxD04. Specifically, DB/DT merged product over ocean is indeed DT.

## 3. Retrieval Algorithm

### 3.1. Retrieval Principal and Sensitivity Analysis

Like the existing DT algorithm, our new algorithm is based on a lookup table (LUT) approach, meaning that it is attempting to match simulated TOA reflectance to the observed TOA reflectance. The best-match solution represents the AOD and other properties of the aerosol. Over the open ocean, the atmospheric signal tends to dominate that of clear water. Near coastlines, however, sediments and turbid waters can dominate the signal, making aerosol retrieval impossible. However, in longer wavelengths, such as 2.1 µm (at which the imaginary part of refractive index for liquid water is several of magnitude larger than that in the visible), the penetration depth is so small, that contribution from the water should be nearly negligible.

**Figure 2:**
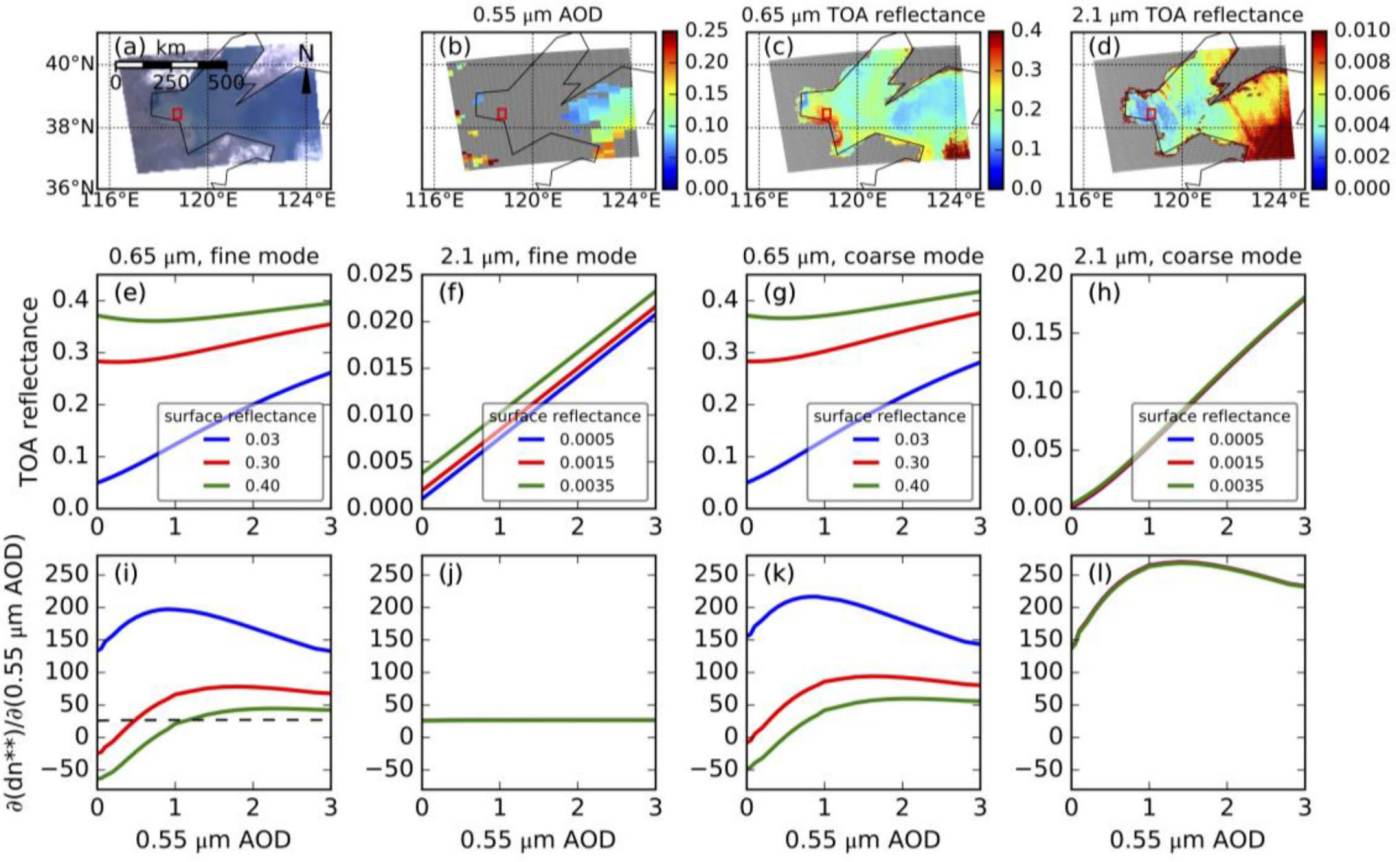
(**a**), (**b**), (**c**), and (**d**) are MODIS Aqua true color image, 0.55 µm AOD, 0.65 µm TOA reflectance, and 2.1 µm TOA reflectance at Bohai Sea, China on Feb. 23, 2017, respectively. Red box is the region where water is turbid and atmosphere is clean. (**e**) and (**f**) are simulation (solar zenith angle is 24°, view zenith angle 54°, and relative azimuth angle 60°) of 0.65 µm and 2.1 µm TOA reflectance with fine aerosol model, respectively. (**g**) and (**h**) are similar to (**e**) and (**f**) but for coarse aerosol model. (**i**) and (**j**) are gradient of 0.65 µm and 2.1 µm MODIS digital signal (dn**) with respect to 0.55 µm AOD with fine aerosol model. (**k**) and (**l**) are similar to (**i**) and (**j**) but for coarse aerosol model. Values in the legends are surface reflectance used to simulate TOA reflectance at corresponding wavelength.

To test this assumption, we compare the sensor sensitivity at 2.1 µm at which water-leaving radiance is negligible regardless of water turbidity with that at shorter wavelength which has large water-leaving radiance over turbid coastal water (taking 0.65 µm as an example). Figure 2 shows 2.1 µm TOA reflectance has better sensitivity to aerosol than 0.65 µm in both fine and coarse mode aerosol situations when 0.65 µm surface reflectance is large (turbid coastal water). When AOD is small (less than 0.15 at 0.55 µm, Figure 2b), the TOA reflectance (Figure 2c,d) is nearly the surface reflectance. Surface reflectance of turbid coastal water is up to 0.4 at 0.65 µm (Figure 2c) while it is very small (less than 0.0035) at 2.1 µm (Figure 2d). Figures 2e–h present a simulation of TOA reflectance through UNL-VRTM model [17] with various surface reflectances in fine aerosol (average TOA reflectance of the four fine aerosol modes defined in the DT-ocean LUT) and coarse aerosol (average TOA reflectance of the coarse aerosol modes defined in the DT-ocean LUT) situations. In a fine aerosol situation, 0.65 µm TOA reflectance is an increasing function of AOD when surface reflectance is small (0.03, clear water) (Figure 2e). However, it first decreases and then increases slowly when surface reflectance is large (0.3 or 0.4, turbid coastal water) (Figure 2e). The gradient of 2.1 µm MODIS digital signal (defined as change of MODIS digital count, *dn* **, with respect to 0.55 µm AOD, or ∂(*dn* **)_2.1_/∂ (*AOD*)_0.55_) is ~25 regardless of AOD values (Figure 2j) while ∂(*dn* **)_0.65_/∂ (*AOD*)_0.55_ is smaller than 25 when AOD is less than 0.5 (1.2) and surface reflectance is 0.3 (0.4). This means that it is better to use TOA reflectance at 2.1 µm rather than 0.65 µm to retrieve fine AOD in turbid coastal water situation. Additionally, while the sensitivity of digital signal at 2.1 µm to the AOD change is a factor of 3–8 smaller than the counterpart at 0.65 µm (e.g., contrasts of blue lines between Figure 2i,j) in the clear water situation, 2.1 µm still has significant sensitivity to the change of fine-mode AOD, and its detection limit for fine-mode AOD is ~0.04 (i.e., the inverse of ∂(*dn* **)_2.1_/∂ (*AOD*)_0.55_). The reason that 2.1 µm still has reasonably good sensitivity for fine-mode aerosols is because the ocean surface is nearly black at 2.1 µm, albeit fine aerosol extinction decrease significantly.

In a coarse aerosol situation, ∂(*dn* **)_0.65_/∂ (*AOD*)_0.55_ is similar to that in a fine aerosol situation and is a little smaller than ∂(*dn* **)_2.1_/∂ (*AOD*)_0.55_ when 0.65 µm surface reflectance is small (Figure 2k,l). However, ∂(*dn* **)_0.65_/∂ (*AOD*)_0.55_ is a factor of 6–10 smaller than ∂(*dn* **)_2.1_/∂ (*AOD*)_0.55_ when water is turbid (e.g., contrast of red/green curves between Figure 2k,l). Overall, over turbid water, it is better to use TOA reflectance at 2.1 µm rather than 0.65 µm to retrieve AOD, regardless of whether fine or coarse mode dominates. In other words, 2.1 µm should be used to retrieve coarse-mode AOD regardless of water turbidity.

### 3.2. Algorithm Implementation and Steps

The retrieval algorithm is designed to supplement the existing DT algorithm. Hence, the lookup table (LUT) from the MODIS DT-ocean AOD retrieval algorithm is also used here [4,9]. That LUT is created by using Ahmad and Fraser’s [18] radiative transfer code, assuming aerosol optical properties from four “fine” (effective radius < 1 µm) and five “coarse” (effective radius > 1 µm) lognormal mode. The fine modes include two water-insoluble and two soluble modes, whereas the coarse modes are separated into three soluble sea-salt like, and two insoluble dust-like modes (http://darktarget.gsfc.nasa.gov/algorithm/ocean/aerosol-models). This LUT is defined at various 0.55 µm AOD values in the range of 0 to 3 and at different Sun-Earth-satellite geometries [4,9]. Furthermore, as water surface reflectance depends on surface wind speed, the LUT is constructed at four wind speeds, specifically 2 m s^−1^, 6 m s^−1^, 10 m s^−1^, and 14 m s^−1^.

Like the DT algorithm, combination of a fine mode and a coarse mode is required for AOD retrieval. In the retrieval procedure, MxD02 TOA reflectance at 2.1 µm is used to fit the corresponding LUT value $\rho_{2.1}^{\text{LUT}}(\tau_{0.55}^{\text{tot}})$ which is a weighted sum of pure fine mode LUT value $\rho_{2.1}^{f}(\tau_{0.55}^{\text{tot}})$ and pure coarse mode LUT $\rho_{2.1}^{c}(\tau_{0.55}^{\text{tot}})$ at a given 0.55 µm AOD value $\tau_{0.55}^{\text{tot}}$.

(1) $$\rho_{2.1}^{\text{LUT}}(\tau_{0.55}^{\text{tot}})=\eta \rho_{2.1}^{f}(\tau_{0.55}^{\text{tot}})+(1-\eta )\rho_{2.1}^{c}(\tau_{0.55}^{\text{tot}})$$

The value of reflectance weighting parameter η equals the fraction of total AOD at 0.55 µm contributed by the fine mode [9]. In addition, the LUT is calculated under the assumption of “gas free” (gas absorption is not included), thus correction [4] of MxD02 TOA reflectance is required to match the LUT before retrieving AOD.

**Figure 3:**
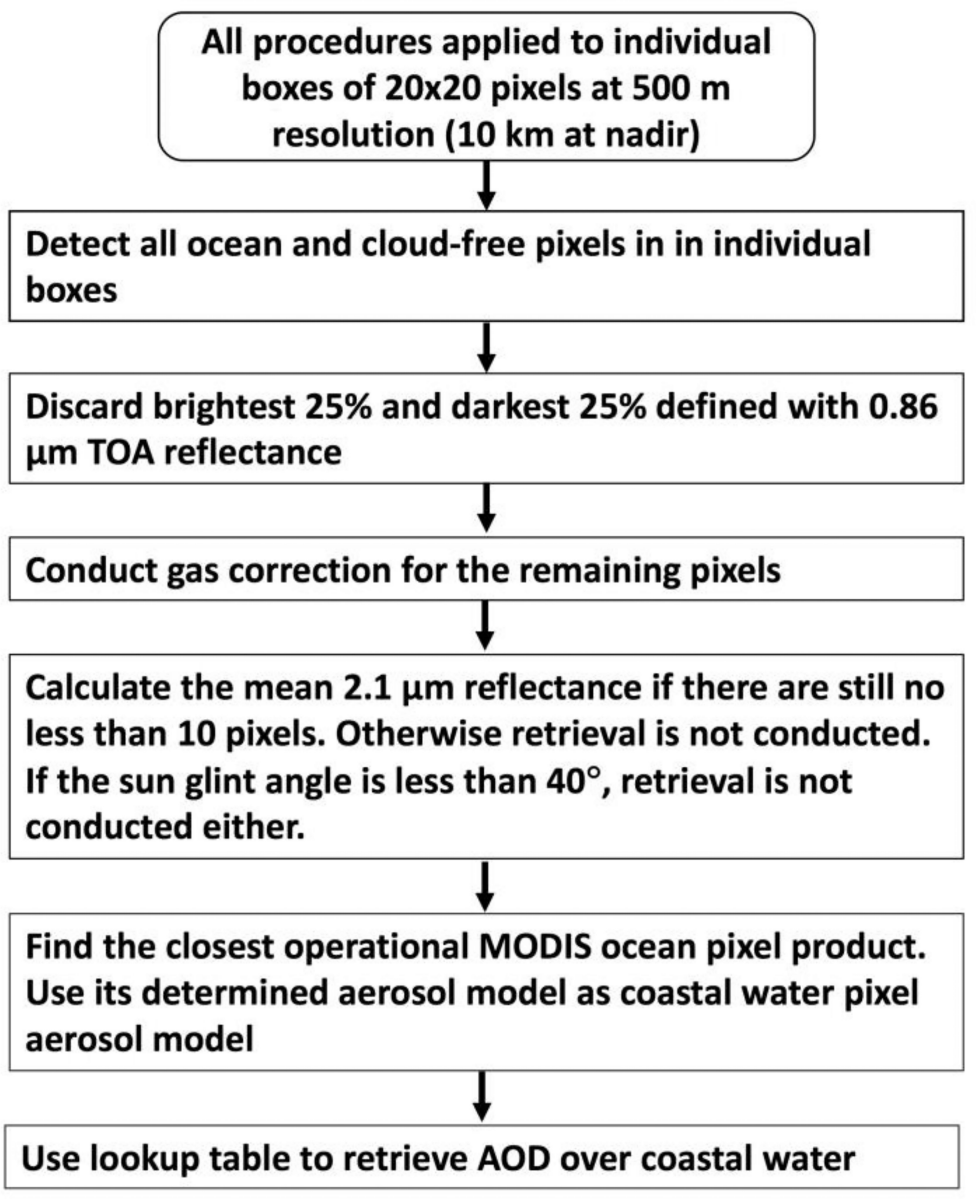
Flowchart of retrieving AOD over coastal water.

Figure 3 shows the steps of our algorithm to retrieve AOD over coastal water, and hereafter, we call this algorithm the Coastal Water (CW) algorithm. Each step of CW is described below with a note that these steps essentially follow DT-Ocean algorithm except CW uses the 2.1 µm to retrieve AOD for those cloud-free turbid coastal water scenes. Hence, for each valid cloud-free turbid coastal water scene (i.e. a box of 20 × 20 pixels at 500 m resolution or at 10 km resolution at nadir, as available in the standard MODIS aerosol product), the following steps are implemented.

**Figure 4:**
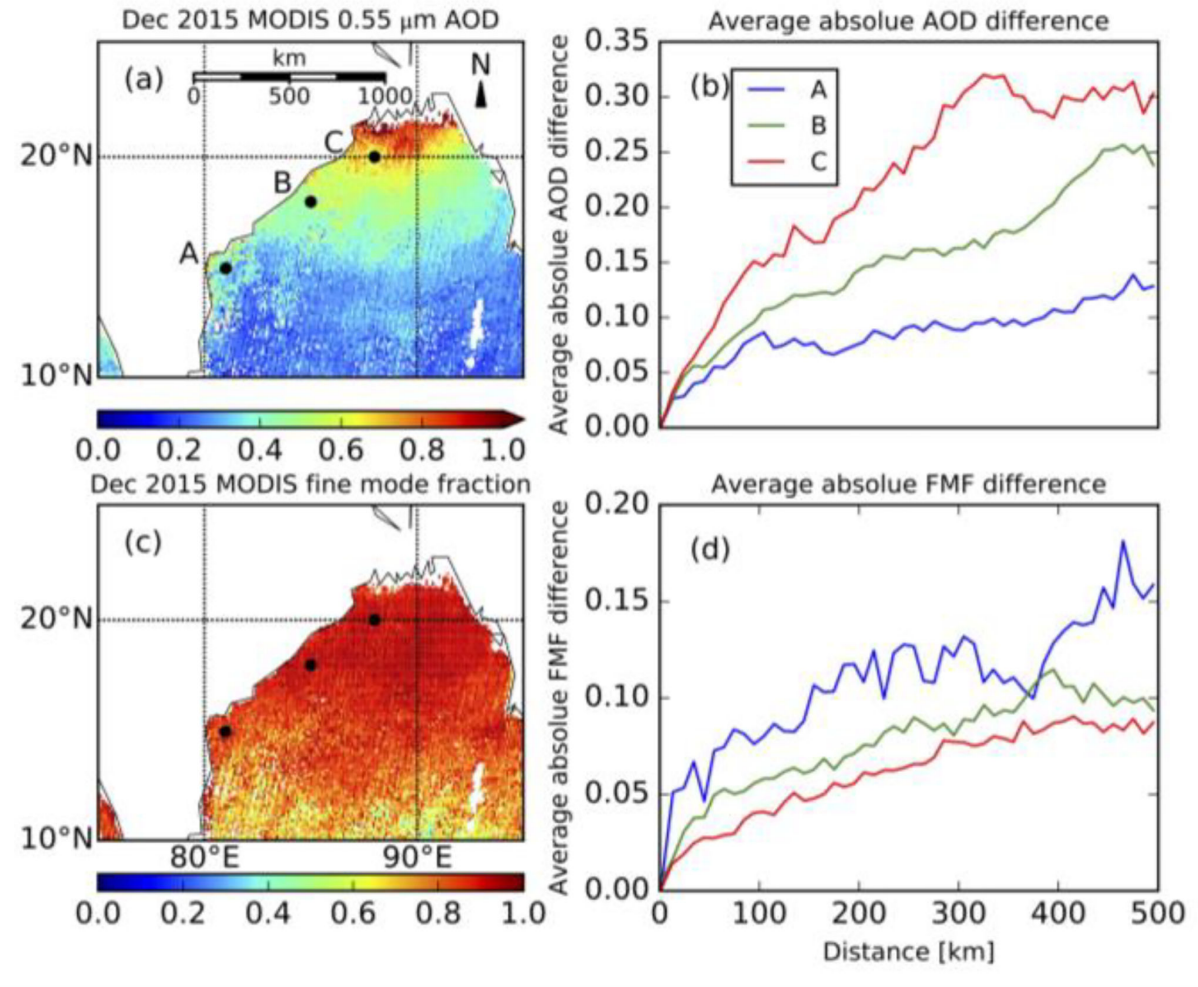
(**a**) and (**c**) are monthly mean 0.55 µm AOD and fine mode fraction (FMF) from MOD04 (Terra) in December, 2015. (**b**) is average absolute AOD difference as a function a distance with respect to reference points (black solid circles in (**a**)). (**d**) is similar to (**b**), but for FMF.

1. Collect and organize 20 × 20 pixels at 500 m resolution, remove pixels that are defined by land/sea mask as “land”, designated by ice/snow mask to be “ice”, designated by the cloud mask to be “cloud”, or removed by other tests.
2. Discard the brightest 25% and darkest 25% pixels defined with 0.86 µm reflectance.
3. Conduct gas (H_2_O, CO_2_, and O_3_) correction [4] for the remaining pixels.
4. Calculate the mean 2.1 µm TOA reflectance if there are still no less than 10 pixels. Otherwise retrieval is not conducted. Calculate the sun glint angle [9]. If the sun glint angle is less than 40°, the retrieval is not conducted.
5. Prescribe single scattering properties of the aerosol. By only using 2.1 µm reflectance to retrieve AOD, there is no sensitivity to aerosol optical properties. Figure 4 shows that AOD can differ up to 0.2 in 100 km from the coast, but FMF differs only by 0.08 in 100 km from the coast. Thus, we assume the single scattering properties (including FMF) and surface wind speed for a turbid coastal water pixel is the same as those used for the AOD retrieval by the standard MODIS algorithm over its closest open-ocean pixel (within 100 km radius). The assumption that aerosol type does not change over moderate spatial scale is reasonable and was used in the atmospheric correction of SeaWiFS imagery over turbid coastal waters [19].
6. Use the mean 2.1 µm TOA reflectance and lookup table determined by Equation (1) to retrieve AOD over the turbid coastal water where MxD04 product is unavailable. In application of Equation (1), all ancillary information (aerosol mode selection and FMF) is obtained from step 5.

## 4. Results

**Figure 5:**
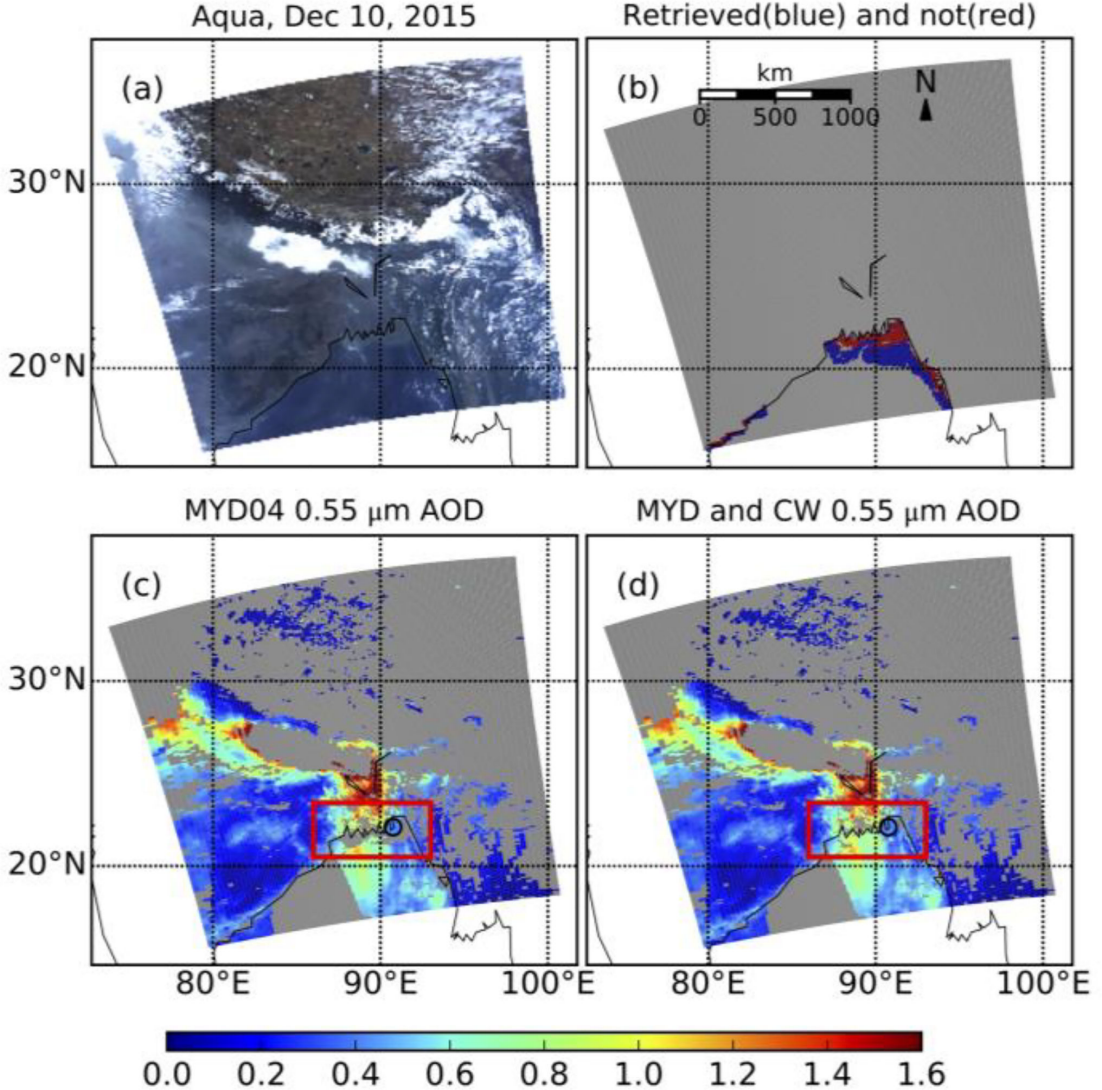
(**a**) Aqua MODIS true color image on 10 December 2015. (**b**) Blue and red represents coastal water where MYD AOD retrievals are available (blue) and unavailable (red), respectively in clear sky condition. (**c**) MYD04 0.55 µm DT/DB merged AOD retrievals. (**d**) is similar to (**c**), but the coastal water gaps where MYD04 AOD retrievals are not available in clear sky condition (red box) are filled by AOD retrievals from CW algorithm. AERONET AOD measurement overlaps on (**c**) and (**d**).

Figure 5 shows the example of CW applied to an Aqua-observed scene (10 December 2015) over the Bay of Bengal. According to the standard DT retrieval (MYD04), AOD reaches 1.6 in the center of the granule and decreases gradually southward. MYD04 does not provide retrievals over the coastal water at north part of the Bay of Bengal (Figure 5c). This non-retrieval region is in cloud-free conditions (Figure 5a), so AOD was not retrieved due to turbid water or underlying sea-floor. Figure 5b shows non-retrievals are in 36.4% of this cloud-free, coastal area (within 5 km of coastline or with water depth less than 50 m).

The CW algorithm enhances DT-ocean, by retrieving AOD over those turbid water conditions (Figure 5d). These “new” retrievals are consistent, in that there appears to be smooth transition in retrieved AOD (e.g., land → CW → ocean). In addition, the new CW AOD retrievals compare well with corresponding values observed by AERONET (overlaid as filled circles in Figure 5c,d).

To validate, we follow the standard protocol, in that AERONET measurements within ±30 min of the MODIS overpass time are averaged and compared against MODIS retrievals averaged within a 25-km radius circular region centered over the AERONET site. This means that there are three situations: MxD04 retrievals only, CW retrievals only, and cases where both are available within the 25-km of the AERONET site.

**Figure 6:**
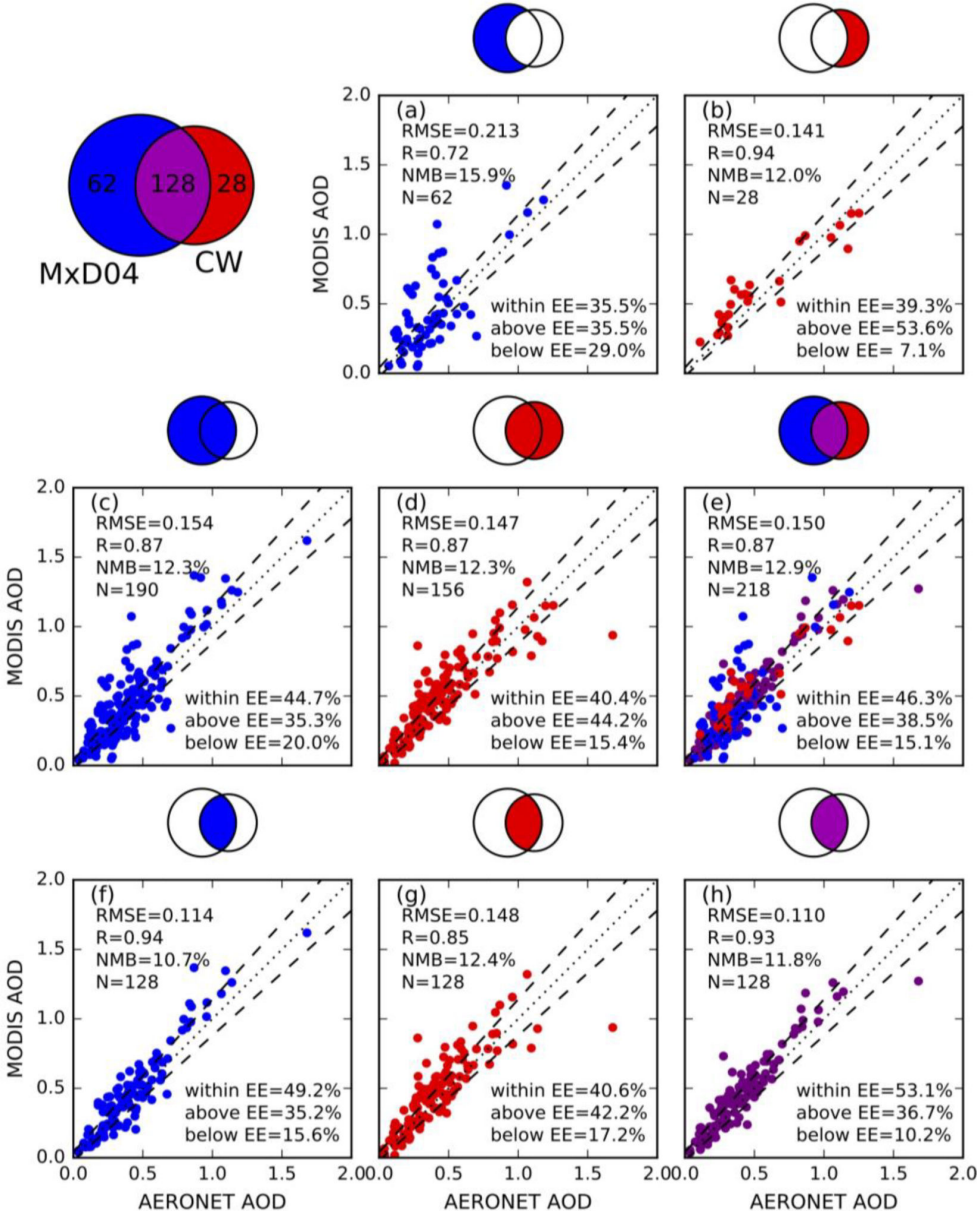
Scatter plots of 0.55 µm AOD retrievals from MODIS (MxD04 and/or CW) versus AERONET observations of 6 sites in one month. Venn diagram on the upper left represents number of collocated data sets for MxD04-AERONET (blue), CW-AERONET (red), (MxD04 + CW)-AERONET (purple). Color part of Venn diagram on the top of each scatter plot (**a–h**) represents which collocated data sets are plotted; see details in the text for further description of the Venn diagram. (**a**) is MxD04-AERONET in the situation that only MxD04 retrievals are available within the 25-km of AERONET site. (**b**) is CW-AERONET in the situation that only CW retrievals are available. (**c**) and (**d**) are similar to (**a**) and (**b**), respectively, but both additionally include AOD retrievals from their respective counterparts of the situation that both MxD04 and CW retrievals are available. (**e**) is (MxD04 + CW)-AERONET in all the three situations. (**f**), (**g**), and (**h**) are MxD04-AERONET, CW-AERONET, (MxD04 + CW)-AERONET, respectively, in the situation that both MxD04 and CW retrievals are available. 1:1 lines and expected error (EE) envelopes (+(0.04 + 10%), −(0.02 + 10%), asymmetric) are plotted as dot and dashed lines. The number of collocated pairs (N), root mean square error (RMSE), normalized mean bias (NMB), and linear correlation coefficient (R) are also shown.

AOD (at 0.55 µm) retrievals from MxD04 product and CW algorithm are evaluated against AERONET data (as shown in Figure 6). We use a Venn diagram in Figure 6 to represent how MODIS AOD retrievals (MxD04 and/or CW) and AERONET observations are collocated. Figure 6a is a scatter plot of MxD04 retrievals versus AERONET measurements in the cases that have retrievals only from MxD04, and these cases can be divided into two categories: (a) all cloud-free pixels within 25-km radius proximity of AERONET site are retrieved through MODIS operational algorithm or (b) only part of cloud-free pixels are retrieved through MODIS DT algorithm, but the AOD of the rest of the cloud-free pixels cannot be retrieved with the CW algorithm because these pixels are over turbid water and no open-ocean pixel is close enough (i.e., within 100 km) to provide aerosol single scattering property for CW algorithm. Figure 6b is a scatter plot of CW retrievals versus AERONET measurements in the cases where all cloud-free pixels within 25-km radius proximity of AERONET site cannot be retrieved through MODIS DT algorithm but some or all of these cloud-free pixels can be retrieved through CW algorithm. Comparison of Figure 6a,b shows that normalized mean bias (NMB, $\text{NMB}=\sum_{i=1}^{N}((\tau_{i}^{\text{MODIS}}-\tau_{i}^{\text{AEROENT}})/\tau_{i}^{\text{AEROENT}})/N)$ and root mean square error (RMSE) of CW algorithm (12.0% and 0.141) are smaller than that of MxD04 (15.9% and 0.213). The percentage of collocated pairs within expected error envelope (+(0.04 + 10%), −(0.02 + 10%), asymmetric) and correlation coefficient increase from 35.5% and 0.72 in MxD04 only situation (Figure 6a) to 39.3% and 0.94 in CW only situation (Figure 6b), respectively.

In addition to the situations where only MxD04 or only CW retrievals are available within the 25-km radius proximity of AERONET site, there is a third one where some of the cloud-free pixels are retrieved through the operational MODIS algorithm while some are retrieved through the CW algorithm. Figure 6c,d are similar to Figure 6a,b, respectively, but both include the AOD retrievals from their respective counterparts of this third situation. Overall, for all possible retrievals by each algorithm, CW retrievals are comparable to MxD04 in quality, although the MODIS operational algorithm has slightly more samples.

The basis of MODIS-AERONET collocation is that air masses are always in motion and the average of MODIS AOD retrievals in a certain area which encompass an AERONET site should be comparable to the temporal statistics of the AERONET measurements [16]. In the situation that both MxD04 and CW retrievals are available within the 25-km radius proximity of AERONET site, if only MxD04 or CW retrievals are collocated with AERONET observation, it will be biased comparison. Therefore, we further show the inter-comparison between either MxD04 or CW AOD with AERONET AOD at this situation (Figure 6f–h). The percentage of collocated pairs in the EE for (MxD04 + CW) combined vs. AERONET (53.1%, Figure 6h) is larger than that of MxD04-AERONET (49.2%, Figure 6f) and CW-AERONET (40.6%, Figure 6g). The RMSE of (MxD04 + CW)-AERONET (0.110) is smaller than that of MxD04-AERONET (0.114) and CW-AERONET (0.148).

Inter-comparison of MxD04 and CW merged AOD with AERONET AOD is shown in Figure 6e; such merged AOD include all data points in Figure 6a,b,h. The number of collocated pairs increases from 190 (62 in MxD04 only situation plus 128 in the situation that both MxD04 and CW retrievals are available) to 218 (28 in CW only situation are added). In addition, the inter-comparison statistics (Figure 6e) is still comparable to or better than those retrieved from MxD04 (Figure 6a,c) in quality. Overall, CW retrievals supplement MODIS DT, and improve the AOD retrievals both spatially and temporally without degrading (and sometimes increasing) the DT-Ocean AOD retrieval quality.

**Figure 7:**
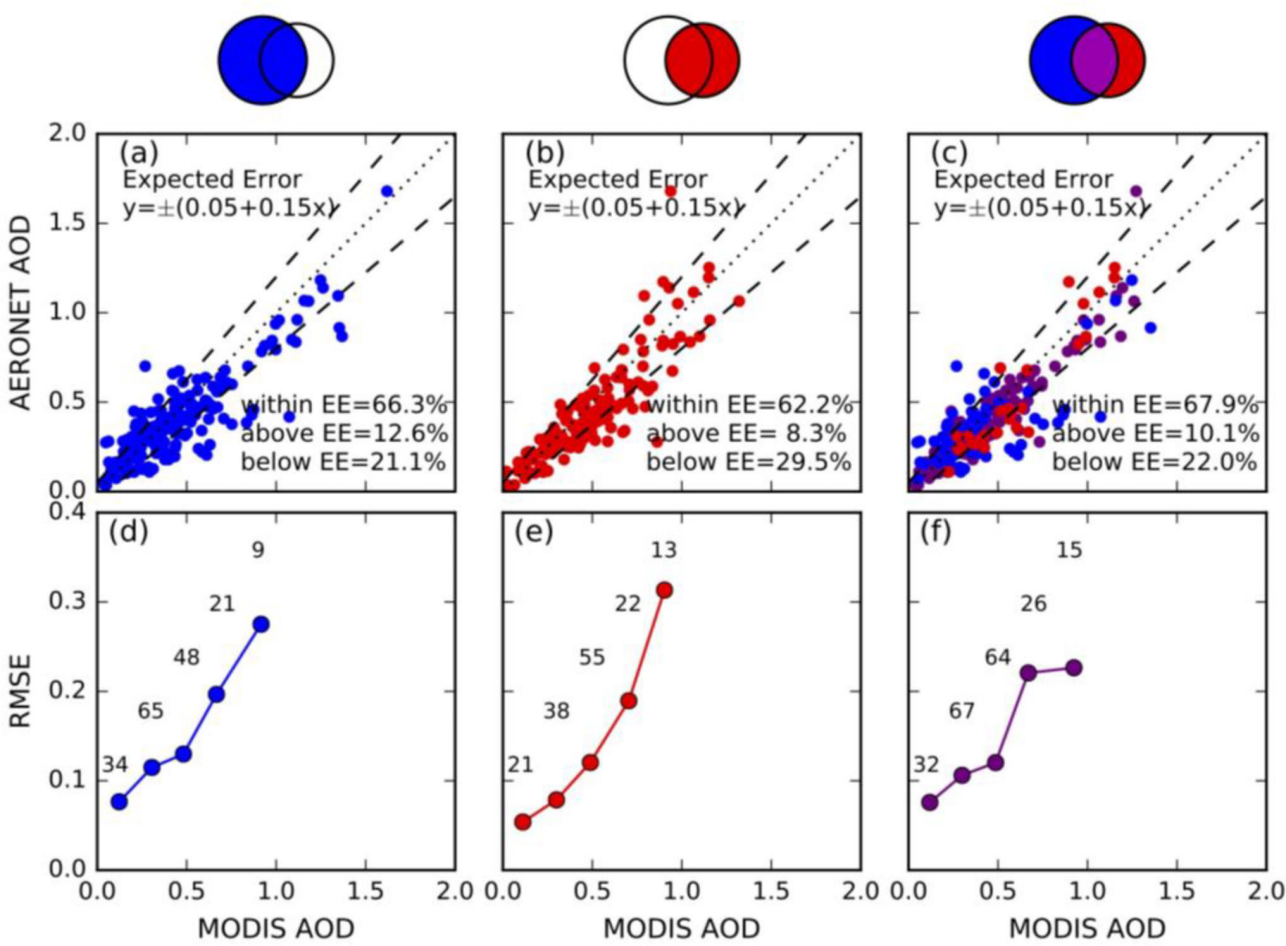
(**a**), (**b**), and (**c**) are scatter plots of AERONET 0.55 µm AOD observations versus retrievals from MODIS (MxD04 and/or CW) for the 6 monthly cases. Sampling method is same as in Figure 6. 1:1 lines and expected error (EE) envelopes (+(0.05 + 15%), −(0.05 + 15%), symmetric) are plotted as dotted and dashed lines. (**d**), (**e**), and (**f**) are root mean square error (RMSE) as a function of MODIS AOD in the 0.2 interval. Numbers above the averaged dot show how many AERONET-MODIS collocations are available to calculate RMSE.

In addition to evaluation of MODIS AOD diagnostic error in Figure 6, prognostic error is presented in Figure 7. Two thirds of AERONET-MODIS collocations are within the expected error envelope (y = ±(0.05 + 0.15x)) (Figure 7c), thus 0.05 + 0.15 (MODIS AOD) can be considered as its prognostic error. Figures 7d–f show RMSE as a function of MODIS; RMSE goes up as MODIS AOD increases and combined retrievals (Figure 7f) are comparable to MxD04 (Figure 7d).

## 5. Discussion

The CW algorithm uses 2.1 µm to retrieve AOD over turbid coastal water as water-leaving radiance is negligible at that band. As only one band is used in the algorithm, aerosol single scattering properties need to be prescribed. We assume the single scattering properties for a turbid coastal water pixel are the same as those used for the AOD retrieval by the standard MODIS algorithm over its closest open-ocean pixel (within 100 km radius). When no pixel is within 100 km, we do not conduct retrieval. Further evaluation can be targeted at the assumptions regarding the use of aerosol single scattering properties from adjacent non-turbid water pixels within certain threshold distance.

The 6 AERONET sites used for this analysis were located in polluted regions. We expect to evaluate how the algorithm performs in an unpolluted zone if there is a new AERONET site or other field campaign that satisfies all the conditions: (1) it is close to coast line; (2) coastal water is turbid; and (3) the region is unpolluted.

The CW algorithm potentially can also be used for the other aerosol retrieval algorithm over the ocean such as the SeaWiFS Ocean Aerosol Retrieval (SOAR) algorithm developed by Sayer et al. [20]. In SOAR, TOA reflectance measured by the SeaWiFS bands centered near 0.51 µm, 0.67 µm, and 0.86 µm are used to retrieve AOD regardless of water turbidity. Hence, as pointed out by Sayer et al. [20], the performance of SOAR retrieval is expected to be poorer in turbidity cases and a double of TOA reflectance as a result of water turbidity causes a positive error in retrieved 0.55 µm AOD of 0.25 [20]. Indeed, in the evaluation of SOAR algorithm, AOD retrievals that are proximity to coast are not included [20].

As MODIS sensors aboard on Terra and Aqua have been providing data for more than one decade and will be decommissioned by the early 2020s, its aerosol record is expected to be continued by the Visible Infrared Imaging Radiometer Suite (VIIRS) on board Suomi-NPP (S-NPP) which was launched in late 2011. Thus, future investigation will also include the application of the approach in this study to VIIRS. VIIRS has 2.26 µm band at which water leaving radiance is also negligible like 2.1 µm band on MODIS and thus the method can be likewise applied.

## 6. Conclusions

The MODIS Dark Target (DT) algorithm has been applied to retrieve AOD over land and ocean since early 2000. Although there have been significant improvements to the algorithm in the past decade, it has not yet been able to retrieve AOD over turbid coastal water due to its high water-leaving radiance from the ocean bottom or water color. We designed the Coastal Water AOD retrieval algorithm (CW algorithm) for these regions to supplement the current DT algorithm. The AOD retrieval algorithm for turbid coastal water takes advantage of the fact that water-leaving radiance is negligible at 2.1 µm, hence this band is only used to retrieve while other auxiliary information such as aerosol single scattering properties are obtained from DT retrievals over the nearby non-turbid water surfaces. In other words, the aerosol modes and reflectance weighting parameter of the pixel to be retrieved is substituted by the closest counterpart from the MxD04 ocean AOD product.

The CW algorithm not only fills the gaps of MxD04 AOD retrievals over turbid coastal water but also improves their comparison with AERONET measurements. CW AOD retrievals are validated against measurements of six AERONET sites that are located at coastal regions. The new algorithm yields ~18% more of MODIS-AERONET collocated pairs, and CW AOD retrievals are comparable or better than MxD04 in quality; the RMSE of MxD04 and CW product is 0.154 and 0.147, respectively. In the situation that both AOD retrievals from MxD04 and CW are available within a 50 km diameter circular regions centered at AERONET sites, the merged AOD retrievals show better agreement with AERONET AOD either of them alone, in terms of the percentage of collocated pairs in the EE (49.2%, 40.6%, and 53.1% for MxD04, CW, and merged product, respectively) and RMSE (0.114, 0.148, 0.110 for MxD04, CW, and merged product, respectively). In addition, 0.05 + 0.15(MODIS AOD) can be considered as prognostic error of the merged product.

## Figures and Tables

**Table 1:** Information of AERONET sites used for validation.

Site	Location *	Period	Data Level	Monthly Mean 0.44–0.87 µm Ångström Exponent
MVCO, New England	41.3°N 70.6°W	August 2015	2.0	1.842
Bhola, Bangladesh	22.2°N 90.8°E	December 2015	1.5	1.206
Anmyon, S. Korea	36.5°N 126.3°E	May 2016	1.5	1.076
Dalma, UAE	24.5°N 52.3°E	August 2004	2.0	0.711
Karachi, Pakistan	24.9°N 67.0°E	March 2014	2.0	0.701
MAARCO, UAE	24.7°N 54.7°E	September 2004	2.0	0.597

* All the locations are shown as lime points in Figure 1f.

## References

[1] Haywood J, Boucher O (2000). Estimates of the direct and indirect radiative forcing due to tropospheric aerosols: A review. Rev. Geophys.

[2] Rosenfeld D, Lohmann U, Raga GB, Dowd CD, Kulmala M, Fuzzi S, Reissell A, Andreae MO (2008). Flood or drought: How do aerosols affect precipitation?. Science.

[3] Lim SS, Vos T, Flaxman AD, Danaei G, Shibuya K, Adair-Rohani H, AlMazroa MA, Amann M, Anderson HR, Andrews KG (2012). A comparative risk assessment of burden of disease and injury attributable to 67 risk factors and risk factor clusters in 21 regions, 1990–2010: A systematic analysis for the global burden of disease study 2010. Lancet.

[4] Levy RC, Mattoo S, Munchak LA, Remer LA, Sayer AM, Patadia F, Hsu NC (2013). The collection 6 modis aerosol products over land and ocean. Atmos. Meas. Tech.

[5] Hsu NC, Jeong MJ, Bettenhausen C, Sayer AM, Hansell R, Seftor CS, Huang J, Tsay SC (2013). Enhanced deep blue aerosol retrieval algorithm: The second generation. J. Geophys. Res.

[6] Sayer AM, Hsu NC, Bettenhausen C, Jeong MJ (2013). Validation and uncertainty estimates for modis collection 6 “deep blue” aerosol data. J. Geophys. Res.

[7] IPCC (2013). Contribution of working group I to the fifth assessment report of the intergovernmental panel on climate change. Climate Change 2013: The Physical Science Basis.

[8] Li R-R, Kaufman YJ, Gao B-C, Davis CO (2003). Remote sensing of suspended sediments and shallow coastal waters. IEEE Trans. Geosci. Remote Sens.

[9] Remer LA, Kaufman YJ, Tanré D, Mattoo S, Chu DA, Martins JV, Li RR, Ichoku C, Levy RC, Kleidman RG (2005). The modis aerosol algorithm, products, and validation. J. Atmos. Sci.

[10] Anderson JC, Wang J, Zeng J, Leptoukh G, Petrenko M, Ichoku C, Hu C (2013). Long-term statistical assessment of aqua-modis aerosol optical depth over coastal regions: Bias characteristics and uncertainty sources. Tellus B.

[11] Wolfe RE, Nishihama M, Fleig AJ, Kuyper JA, Roy DP, Storey JC, Patt FS (2002). Achieving sub-pixel geolocation accuracy in support of modis land science. Remote Sens. Environ.

[12] King MD, Menzel WP, Kaufman YJ, Tanre D, Bo-Cai G, Platnick S, Ackerman SA, Remer LA, Pincus R, Hubanks PA (2003). Cloud and aerosol properties, precipitable water, and profiles of temperature and water vapor from modis. IEEE Trans. Geosci. Remote Sens.

[13] Smirnov A, Holben BN, Eck TF, Dubovik O, Slutsker I (2000). Cloud-screening and quality control algorithms for the aeronet database. Remote Sens. Environ.

[14] Holben BN, Eck TF, Slutsker I, Tanré D, Buis JP, Setzer A, Vermote E, Reagan JA, Kaufman YJ, Nakajima T (1998). Aeronet—A federated instrument network and data archive for aerosol characterization. Remote Sens. Environ.

[15] O’Neill NT, Eck TF, Smirnov A, Holben BN, Thulasiraman S (2003). Spectral discrimination of coarse and fine mode optical depth. J. Geophys. Res.

[16] Ichoku C, Chu DA, Mattoo S, Kaufman YJ, Remer LA, Tanré D, Slutsker I, Holben BN (2002). A spatio-temporal approach for global validation and analysis of modis aerosol products. Geophys. Res. Lett.

[17] Wang J, Xu X, Ding S, Zeng J, Spurr R, Liu X, Chance K, Mishchenko M (2014). A numerical testbed for remote sensing of aerosols, and its demonstration for evaluating retrieval synergy from a geostationary satellite constellation of geo-cape and goes-r. J. Quant. Spectrosc. Radiat. Transf.

[18] Ahmad Z, Fraser RS (1982). An iterative radiative transfer code for ocean-atmosphere systems. J. Atmos. Sci.

[19] Hu C, Carder KL, Muller-Karger FE (2000). Atmospheric correction of seawifs imagery over turbid coastal waters: A practical method. Remote Sens. Environ.

[20] Sayer AM, Hsu NC, Bettenhausen C, Ahmad Z, Holben BN, Smirnov A, Thomas GE, Zhang J (2012). Seawifs ocean aerosol retrieval (soar): Algorithm, validation, and comparison with other data sets. J. Geophys. Res. Atmos.

